# Supplementary figures and images for: Acupuncture Decreases Risk of Hypertension in Patients with Chronic Spontaneous Urticaria in Taiwan: A Nationwide Study
Source: Healthcare (Basel). 2023 May 22;11(10):1510. doi: 10.3390/healthcare11101510 (PMC10218557; doi:10.3390/healthcare11101510)

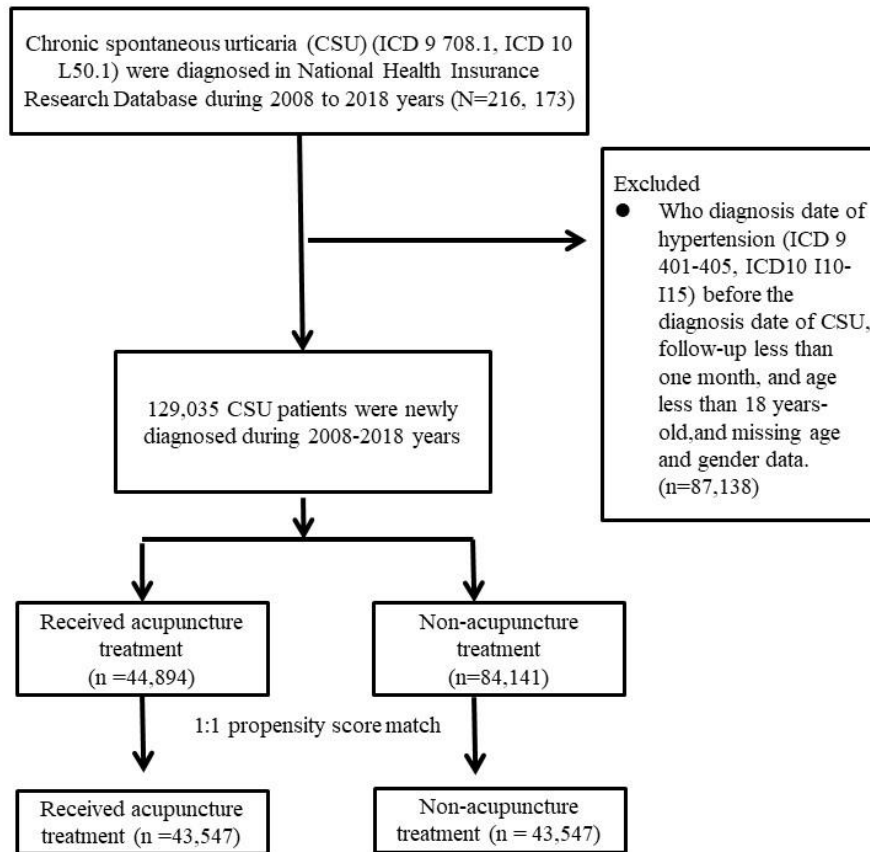

Figure S1: flow chart.

Supplement: Supplementary file 1 [file healthcare-11-01510-s001.zip › healthcare-2375330-supplementary.pdf]
